# Frailty as a Predictor of In-Hospital Outcome in Patients with Myocardial Infarction

**DOI:** 10.3390/jcdd9050145

**Published:** 2022-05-05

**Authors:** Michał Węgiel, Paweł Kleczyński, Artur Dziewierz, Łukasz Rzeszutko, Andrzej Surdacki, Stanisław Bartuś, Tomasz Rakowski

**Affiliations:** 1 Department of Cardiology and Cardiovascular Interventions, University Hospital, 30-688 Krakow, Poland; michal.jan.wegiel@gmail.com; 2 Department of Interventional Cardiology, Institute of Cardiology, Jagiellonian University Medical College, 31-202 Krakow, Poland; kleczu@interia.pl; 3 Second Department of Cardiology, Institute of Cardiology, Jagiellonian University Medical College, 30-688 Krakow, Poland; adziewierz@gmail.com (A.D.); rzeszutko.lukasz@gmail.com (Ł.R.); andrzej.surdacki@uj.edu.pl (A.S.); stanislaw.bartus@uj.edu.pl (S.B.)

**Keywords:** myocardial infarction, frailty, delirium, pneumonia, hospitalization length

## Abstract

(1) Background: Frailty is a condition associated with aging, co-morbidity, and disability. We aimed to investigate the relationship between frailty and in-hospital outcome in patients with myocardial infarction (MI), including the occurrence of delirium, hospital-acquired pneumonia (HAP), and length of hospital stay. (2) Methods: We analyzed 55 patients ≥ 75 years old with ST-elevation and non-ST-elevation MI. Assessment with Abbreviated Mental Test Score (AMTS), Activity of Daily Living (ADL), Instrumental Activity of Daily Living (IADL) and Clinical Frailty Scale (CFS) was performed. (3) Results: In ROC analysis, IADL and CFS presented good predictive values for the occurrence of delirium (AUC = 0.81, *p* = 0.023, and AUC = 0.86, *p* = 0.009, respectively). For predicting HAP, only AMTS showed a significant value (AUC = 0.69, *p* = 0.036). In regression analyses, all tests presented significant predictive values for delirium. For predicting HAP, only IADL and CFS presented significant values (in an analysis adjusted for age, gender and type of MI). Frail patients (≥5 points in CFS) had longer hospital stays (10 days IQR: 8–17 vs. 8 days IQR: 7–10; *p* = 0.03). (4) Conclusions: While recognizing the limitations of our study associated with the relatively low sample size, we believe that our analysis shows that frailty is a predictor of poorer in-hospital outcomes in patients with MI, including higher rates of delirium, HAP and longer hospital stay.

## 1. Introduction

Frailty is a condition associated with aging, co-morbidity, and disability [1,2]. It includes both mental and physical deterioration. The occurrence of frailty may aggravate the course of illnesses, including myocardial infarction (MI) [1,2]. In the aging population, frailty is becoming a frequent issue in patients hospitalized with MI. It worsens the treatment outcome and causes prolonged hospital stays [3,4]. Frailty has been correlated with greater mortality and higher rates of bleeding and cerebrovascular events. However, there are little data about the impact of frailty on the occurrence of delirium and pneumonia in patients hospitalized with MI. The aim of the presented study was to assess the daily activity and cognitive status of elderly patients with MI and evaluate the relationship between impairment in those areas and in-hospital outcomes.

## 2. Materials and Methods

The present study is a prospective registry focused on frailty assessment in patients hospitalized with MI. The study was approved by the local ethics committee at the Jagiellonian University Medical College University (number 122.6120.2.2017). All procedures involving study subjects were performed according to the principles published by the declaration of Helsinki and its amendments. Inclusion criteria for entering the study were as follows: ST-segment elevation MI (STEMI) or non-ST-segment elevation MI (NSTEMI) and age ≥ 75 years old. Exclusion criteria were as follows: hemodynamic instability and critical state during hospitalization. Serial 12-lead electrocardiogram (ECG) recordings were obtained according to the local protocol (Mortara Instruments Inc., Milwaukee, USA). During the hospital stay, transthoracic echocardiography examinations were performed using CX Ultrasound System (Phillips, Netherlands). Typical biochemical measurements of cardiac troponin, electrolytes and blood morphology were obtained. We assessed demography, baseline clinical characteristics, medical management (invasive vs. conservative treatment), administered pharmacotherapy, in-hospital outcome including mortality, bleeding complications (defined as a need for red blood cell transfusion) and cerebrovascular events as well as the length of hospital stay, occurrence of delirium, and hospital-acquired pneumonia (HAP). Delirium was defined as an acute worsening of mental status, requiring sedative drugs. HAP was defined as clinical symptoms of respiratory tract infections or typical changes in chest X-ray and treatment with antibiotics, occurring after 48 h from admission.

In the present study, we used the following questionnaires: Abbreviated Mental Test Score (AMTS), Activity of Daily Living (ADL), Instrumental Activity of Daily Living (IADL) and Clinical Frailty Scale (CFS) [5,6,7,8]. AMTS focuses on cognitive function and the patient is scored 0 to 10 points. ADL investigates elementary activities such as eating, using the toilet, and getting dressed and the score ranges from 0 to 6. IADL focuses on the ability to perform more complex tasks such as using a phone, preparing meals, managing finances and medications and the score ranges from 0 to 24. CFS is a graphical/descriptive tool to assess frailty. The score ranges from I to IX as follows: I—very fit; II—fit; III—managing well; IV—vulnerable; V—mildly frail; VI—moderately frail; VII—severely frail; VIII—very severely frail; IX—terminally ill. The assessment was performed at a consistent interval during hospitalization. We aimed at testing during the first 48 h from admission; however, we avoided conducting questionnaires directly after coronary angiography, on the first day of hospitalization or during temporal deterioration of a medical condition. 

Quantitative variables were described using means and standard deviation (for normal distribution of data) or median with the first and the third quartile (for non-normal distribution of data). Normality was assessed by the Shapiro–Wilk test. The Wilcoxon test was used for comparing data. Categorical variables were presented as percentages. A chi-square test was used for comparing categorical data. Receiver operating characteristics (ROC) curves with area under the curve (AUC) and simple logistic regression analyses (adjusted and unadjusted) were performed to identify the predictive value of assessed questionnaires for the occurrence of delirium and HAP. The results are presented as odds ratios with an associated 95% confidence interval. The level of statistical significance was set at alpha value <0.05. 

## 3. Results

**Figure 1:**
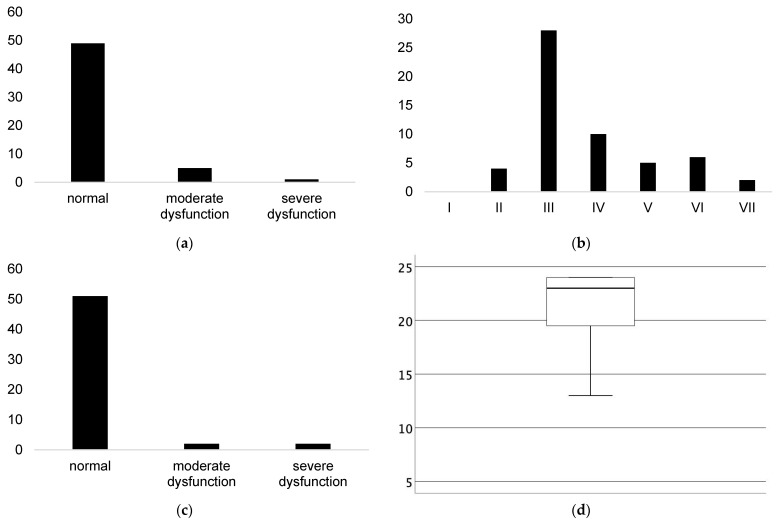
Distribution of results of performed questionnaires presented as the number of patients. (**a**) Abbreviated Mental Test Score; (**b**) Clinical Frailty Scale; (**c**) Activity of Daily Living; (**d**) Instrumental Activity of Daily Living. Results of Instrumental Activity of Daily Living are presented as a box-plot since there are no established cut-off points for defining dysfunction in this test.

Fifty-five patients entered the study. In Table 1, we present study group characteristics including demography, cardiovascular risk factors, results of laboratory tests and performed questionnaires. Men were more common than women in our study and the most common clinical presentation was NSTEMI. In Figure 1, we present the distribution of results of performed questionnaires. At least moderate impairment in cognitive status (AMTS < 7 points) and in daily activity (ADL < 5 points) was found in 9% and 4% of patients, respectively. On the other hand, mild frailty (CFS ≥ 5 points) was found in 24% of patients.

In Table 2, we present characteristics of in-hospital course and outcome. Almost all patients underwent coronary angiography. Most of them were treated with primary percutaneous coronary intervention (PCI). A definite minority of patients were referred to coronary artery bypass grafting (CABG). Administered pharmacotherapy is presented in Table 2. Most patients were treated with clopidogrel instead of more potent P2Y_12_ inhibitors. Delirium occurred in 9% of patients and HAP was diagnosed in 24% of patients. Rates of in-hospital mortality, cerebrovascular events, RBC transfusions and length of hospital stay are demonstrated in Table 2.

In Table 3, we show ROC analyses for predicting the occurrence of delirium and HAP. AMTS, IADL and CFS presented significant predictive value for predicting delirium. Out of the examined questionnaires, only AMTS had significant value for predicting HAP. 

In regression analyses all evaluated tests presented predictive value for delirium. On the other hand, in an unadjusted analysis none of the examined tests reached statistical significance for predicting HAP. In an analysis adjusted for age, gender and type of MI IADL and CFS presented significant predictive value for occurrence of HAP (Table 4). Patients with at least moderate dysfunction in AMTS (<7 points) more often experienced delirium (50% vs. 2%; *p* = 0.004) and had longer hospital stay (11 days Q1:9 Q3:15 vs. 8 days Q1:7 Q3:10; *p* = 0.046). Patients with at least mild frailty (fifth class in CFS scale and beyond) also had longer hospital stay (10 days Q1:8 Q3:17 vs. 8 days Q1:7 Q3:10; *p* = 0.03). 

## 4. Discussion

The most important finding from our work is that frailty is a relatively frequent phenomenon in patients hospitalized with MI and is a predictor of poorer in-hospital outcomes including higher rates of delirium, pneumonia and longer hospital stay. The term frailty includes impairment in cognitive status, physical general condition, ability to complete daily activities, and vulnerability to external factors. As the general population is becoming older, also patients with MI are more frequently elderly and with many comorbidities [1,2]. Frailty is becoming another factor that impacts the management and outcome of patients with MI [9]. The rate of patients who underwent coronary angiography in our study was very high. However, despite performed angiography, circa 20% of patients were treated conservatively. Previous studies showed that frail patients are less likely to be treated invasively with primary PCI [4,10]. On the other hand, registries are showing the benefit of invasive treatment, even in frail patients with MI [11]. This population is typically insufficiently represented in clinical trials and the management is based on local protocols and consensuses. Furthermore, previous reports showed that type 2 MI is frequent in this group of patients and age-related conditions may be triggering factors for the occurrence of this type of MI [12]. In the presented study, most patients were treated with clopidogrel instead of more potent P2Y_12_ inhibitors. This observation could reflect the concern about higher bleeding risk in this vulnerable population. Frailty has been associated with higher in-hospital and long-term mortality and higher re-hospitalization rates and has been correlated with poorer in-hospital outcomes, including cerebrovascular and bleeding events [1,2,3,4,11,13,14]. 

In previous reports, frailty has been associated with a higher risk of pneumonia and hospitalization in a general population of older adults [15]. There are little data about the impact of frailty on the occurrence of HAP in patients with MI. Our study suggests that frailty is also a risk factor for HAP in those patients. A probable explanation might be that frail patients could be more vulnerable to choking, less eager for physical rehabilitation and want to stay in bed longer. 

Another assessed issue is the occurrence of delirium in patients with MI. Delirium is a relatively common condition in elderly patients with MI treated invasively and has been associated with higher in-hospital mortality and increased length of hospital stay [16,17]. In our analysis, initial impairment of both cognitive status and daily activity were predictors of delirium.

The presented study has several limitations. First of all, the sample size is small with relatively low rates of events. The observation is limited only to the index hospitalization time, with no follow-up data beyond discharge available. Secondly, both STEMI and NSTEMI patients were included in the analysis. This could provide a bias in obtained results, since management of ischemia may differ in both groups of patients. Due to the small sample size, we did not perform separate analyses for both types of MI. Thirdly, the study was performed in a single center which is an interventional cardiology department with a cath lab on-site; thus, almost all patients underwent coronary angiography. In remote centers, the situation might be different and many older, frail patients with NSTEMI might be disqualified from invasive management. On the other hand, previous reports showed that contrast injection could be a triggering factor for delirium [18]. This observation could attribute to the relatively high rate of delirium in the present study. Finally, in our study, delirium was defined as an acute disturbance in mental status and need for sedative/antipsychotic drugs and was diagnosed on the basis of subjective, general examination. We did not perform a quantitative assessment for the diagnosis or monitoring of delirium.

## 5. Conclusions

Frailty seems to be a predictor of poorer in-hospital outcomes in patients with MI, including longer hospital stays, the occurrence of hospital-acquired pneumonia and delirium. Yet, these results should be confirmed in studies with a greater sample size.

## Figures and Tables

**Table 1:** Study group characteristics.

Age (years)	82 ± 5
Male gender (%)	56
ST-elevation MI (%)	24
Non-ST-elevation MI (%)	76
Diabetes mellitus (%)	38
Arterial hypertension (%)	93
Smoking (%)	27
Chronic obstructive pulmonary disease (%)	9
History of PCI (%)	27
History of MI (%)	29
History of CABG (%)	7
History of stroke (%)	9
LVEF (Q1; Q3)	45 (30; 50)
BMI (kg/m^2^)	27.5 ± 4
BSA (m^2^)	1.84 ± 0.18
Hemoglobin (g/dL)	12.9 ± 1.65
White blood count (10^3^/µL)	10.8 ± 3.9
Platelet count (10^3^/µL)	239 ± 81
Glomerular filtration rate (ml/min/1.73 m^2^)	60.3 ± 18.9
Abbreviated Mental Test Score (Q1; Q3)	10 (8, 10)
Activity of Daily Living (Q1; Q3)	6 (6, 6)
Instrumental Activity of Daily Living (Q1; Q3)	23 (19, 24)
Clinical Frailty Scale (Q1; Q3)	3 (3, 4)

MI-myocardial infarction; PCI-percutaneous coronary intervention; CABG-coronary artery bypass grafting; LVEF-left ventricle ejection fraction; BMI-body mass index; BSA-body surface area.

**Table 2:** In-hospital course and outcome.

Coronary angiography (%)	98
Treatment with PCI (%)	72
Treatment with CABG (%)	4
ASA (%)	96
Clopidogrel (%)	74
Ticagrelor (%)	18
Prasugrel (%)	0
B-blocker (%)	94
Angiotensin converting enzyme inhibitor (%)	87
Statin (%)	96
Mortality (%)	2
Stroke (%)	0
Red blood cell transfusion (%)	2
Delirium (%)	9
Hospital acquired pneumonia (%)	24
Length of hospital stay (days) (Q1; Q3)	8 (7; 12)

ASA-acetylsalicylic acid; other abbreviations as in Table 1.

**Table 3:** ROC analysis for the occurrence of delirium and hospital-acquired pneumonia.

	AUC	*p*
**Delirium**		
Activity of Daily Living	0.76	0.052
Instrumental Activity of Daily Living	0.81	0.023
Clinical Frailty Scale	0.86	0.009
Abbreviated Mental Test Score	0.78	0.04
**Pneumonia**		
Activity of Daily Living	0.49	0.94
Instrumental Activity of Daily Living	0.67	0.065
Clinical Frailty Scale	0.62	0.19
Abbreviated Mental Test Score	0.69	0.036

AUC-area under the curve.

**Table 4:** Logistic regression analyses for predicting the occurrence of hospital-acquired pneumonia and delirium.

	Unadjusted Analysis			Analysis Adjusted for Age, Gender and Type of MI		
	OR	95% CI	*p*	OR	95% CI	*p*
**Delirium**						
Activity of Daily Living	0.45	0.23–0.88	0.02	0.32	0.12–0.85	0.022
Instrumental Activity of Daily Living	0.8	0.65–0.97	0.023	0.75	0.58–0.96	0.025
Clinical Frailty Scale	2.63	1.23–5.6	0.012	6.4	1.49–27.4	0.013
Abbreviated Mental Test Score	0.59	0.4–0.88	0.009	0.57	0.36–0.88	0.013
**Pneumonia**						
Activity of Daily Living	1.19	0.56–2.56	0.65	0.92	0.39–2.17	0.85
Instrumental Activity of Daily Living	0.89	0.78–1.02	0.11	0.8	0.66–0.97	0.021
Clinical Frailty Scale	1.35	0.84–2.16	0.21	2.7	1.23–5.9	0.013
Abbreviated Mental Test Score	0.77	0.57–1.04	0.08	0.75	0.55–1.03	0.08

OR-odds ratio, CI-confidence interval.

## Data Availability

Data is contained within the article.
